# Five-Cavity Resonance Inspired, rGO Nano-Sheet Reinforced, Multi-Site Voice Synergetic Detection Hydrogel Sensors with Diverse Self-Adhesion and Robust Wireless Transmissibility

**DOI:** 10.3390/gels11040233

**Published:** 2025-03-23

**Authors:** Yue Wu, Kewei Zhao, Jingliu Wang, Chunhui Li, Xubao Jiang, Yudong Wang, Xiangling Gu

**Affiliations:** 1 College of Chemistry and Chemical Engineering, University of Jinan, Jinan 250024, China; wy990525@163.com (Y.W.); wlljyo12@163.com (J.W.); 2 School of Health and Medicine, Dezhou University, Dezhou 253023, China; 17662433968@163.com (K.Z.); lichunhui-tj@163.com (C.L.); 3 College of Chemistry and Chemical Engineering, Shandong University of Technology, Zibo 255000, China; 4 College of Biological and Chemical Engineering, Guangxi University of Science and Technology, Liuzhou 545006, China

**Keywords:** hydrogel, flexible sensor, five cavities, synergetic enhancement, voice detection

## Abstract

The practical application of flexible sensors in sound detection is significantly hindered by challenges such as information isolation, fragmentation, and low fidelity. To address these challenges, this work developed a composite hydrogel via a one-pot method, employing polyvinyl alcohol (PVA) as the first network, polyacrylic acid (PAA) as the second network, and two-dimensional nanomaterials—reduced graphene oxide (rGO)—generated through the redox reaction of polydopamine (PDA) and graphene oxide (GO) as conductive fillers. The uniformly distributed rGO within the hydrogel forms an efficient conductive network, endowing the material with high sensitivity (GF = 0.64), excellent conductivity (8.15 S m^−1^), rapid response time (350 ms), and outstanding stability. The synergistic interaction between PDA and PAA modulates the hydrogel’s adhesion (0.89 kPa), enabling conformal attachment to skin surfaces. The designed rGO@PVA-PAA hydrogel-based flexible sensor effectively monitors vibrations across diverse frequencies originating from five vocal cavities (head, nasal, oral, laryngeal, and thoracic cavities) during singing. Integrated with multi-position synchronization and Bluetooth wireless sensing technologies, the system achieves coordinated and efficient monitoring of multiple vocal cavities. Furthermore, the hydrogel sensor demonstrates versatility in detecting physiological signals, including electrocardiograms, subtle vibrations, and multi-scale body movements, highlighting its broad applicability in biomedical and motion-sensing applications.

## 1. Introduction

Real-time monitoring of physiological signals provides an effective means for people to maintain their health [1]. Sound, as the most critical communication medium in daily life, not only fulfills the function of information exchange but also represents a vital physiological signal for disease diagnosis and treatment [2]. The sound generated by the vibration of human vocal organs is characterized by high frequency and low amplitude, and this unique acoustic signal can serve as a diagnostic basis for various diseases [3]. For example, respiratory diseases such as pneumonia and asthma can be assessed through voice or cough sounds [4]. Early-stage speech disorders in Parkinson’s patients can be identified via voice pattern recognition [5]. Deep learning techniques applied to respiratory sounds enable effective detection of chronic obstructive pulmonary disease, significantly improving diagnostic accuracy [6].

The resonance of the five vocal cavities plays a crucial role in both singing and everyday vocal production, which refer to the head cavity, nasal cavity, oral cavity, laryngeal cavity, and thoracic cavity [7]. By reasonably adjusting the state of each vocal cavity, a more diverse and more moving sound can be achieved [8,9]. Based on the synergistic detection of multiple vocal cavities, it is possible to obtain a more precise, three-dimensional, and diversified understanding of the vocalizer’s health status, and provide first-hand information for vocal skill improvement or diagnosis and treatment [10]. However, conventional acoustic sensors are prone to environmental interference, and acoustic signals are often attenuated during propagation, leading to uncertainties and errors in data monitoring. In this context, wearable sensors demonstrate groundbreaking advantages by capturing signals directly at the source through skin contact, thereby circumventing environmental noise and ensuring signal integrity [11,12,13].

Rigid sensors fabricated from traditional metals or silicon fail to meet the requirements for monitoring complex physiological signals within the human body [14]. Consequently, the combination of elastomers and conductive fillers has been widely employed in flexible sensors [15,16,17]. However, the low flexibility of conductive elastomers and their poor adhesion to biological tissues severely limit their practical applications. Hydrogels, as soft materials, are regarded as promising candidates for flexible wearable devices due to their excellent flexibility and biocompatibility [18,19,20]. Polyvinyl alcohol (PVA), a water-soluble polymer with remarkable hydrophilicity, biocompatibility, and degradability, is an ideal eco-friendly material for hydrogel preparation. The abundant hydroxyl groups on its molecular chains facilitate the formation of physical or chemical crosslinked networks [21,22]. Nevertheless, the inferior mechanical properties of PVA hydrogels significantly restrict their operational environments. To address this, researchers have enhanced mechanical performance by incorporating reinforcing materials [23,24,25] or constructing multi-crosslinked networks [26,27]. However, the resulting hydrogels often exhibit insufficient electrical conductivity. Furthermore, the ability of hydrogel sensors to conformally adhere to the skin is critical for reliable signal acquisition. Therefore, developing a flexible hydrogel sensor with ultrahigh sensitivity, high fidelity, signal discriminability, and skin-conforming adhesion for multi-site acoustic monitoring holds significant importance.

To address this challenge, a composite hydrogel was synthesized using a one-pot method, with PVA as the first network, polyacrylic acid (PAA) as the second network, and two-dimensional nanosheet reduced graphene oxide (rGO) as the conductive filler from the redox reaction of polydopamine (PDA) and graphene oxide (GO) to improve both the dispersion of nanomaterials and the conductivity of the hydrogel. Due to the structural similarity between PDA and mussel proteins, the synergistic effect of PDA and PAA is able to enhance the adhesiveness of the hydrogel, making it suitable for skin adhesion [28]. To address the specific requirements of high frequency and low amplitude for voice detection, rGO is uniformly distributed within the hydrogel, which not only ensures remarkable adhesion of the hydrogel but also forms an efficient conductive network, enabling the rGO@PVA-PAA hydrogel-based flexible sensor to meet the needs of monitoring different frequency vibrations in the five vocal cavities during human vocalization. Multi-channel and Bluetooth wireless sensing technology are employed to achieve synergistic and efficient detection of multiple vocal cavities in the human body. Additionally, the potential of the fabricated hydrogel sensor as a flexible probe for detecting various physiological signals, including electrocardiograms and even subtle vibrations or large-scale movements that may be difficult for the human body to perceive, is also explored.

## 2. Results and Discussion

### 2.1. Preparation and Characteristic of rGO@PVA-PAA

**Figure 1:**
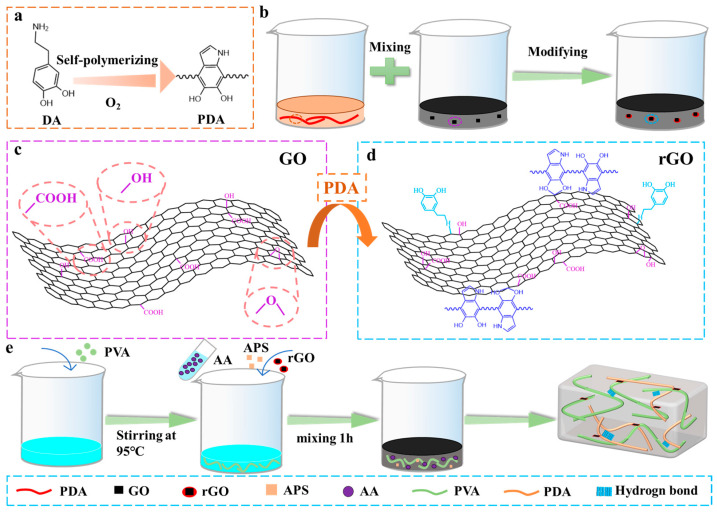
(**a**) Self-polymerization of DA to form a PDA structure diagram; (**b**) flow chart of PDA reduction of GO; (**c**) GO and (**d**) rGO surface modification schematics; (**e**) schematic diagram of the preparation of rGO@PVA-PAA hydrogel.

Figure 1a shows the schematic illustration of the formation of rGO with PDA as a reducing agent. GO is uniformly dispersed in deionized water by ultrasound. DA powder is placed in an open vessel containing Tris-HCl buffer solution (pH 8.5) for stirring to ensure that DA can fully contact O_2_ to form PDA. As the color of the solution changes from transparent to dark brown, Subsequently, when the PDA solution is uniformly mixed with the GO solution, GO undergoes a reduction reaction with the PDA, as shown in Figure 1b. This process removes some oxygen-containing functional groups from GO, reducing it to rGO as shown in Figure 1c,d. The catechol groups on PDA particles modify the surface of rGO, providing more interaction sites [29]. PDA serves as a reducing agent for GO [30], and the rGO prepared through this reaction is expected to enhance the electrical conductivity of composites while mitigating the adverse effects of high concentrations of conductive fillers on material mechanics [31,32].

PAA can be polymerized using AA in the presence of ammonium persulfate (APS) at 70 °C, which offers the advantage of strong adhesion, making it one of the commonly used adhesives in bonding applications. Its adhesion mechanism primarily relies on the establishment of hydrogen bonds, which is attributed to the high -COOH content in PAA [33]. Nevertheless, the low mechanical strength of PAA hydrogels limits their applications in flexible sensors. PVA contains abundant -OH groups that can form hydrogen bonds with the -COOH groups of PAA. The construction of a double-network structure using PVA and PAA significantly enhances the mechanical properties of hydrogels (Figure 1e).

**Figure 2:**
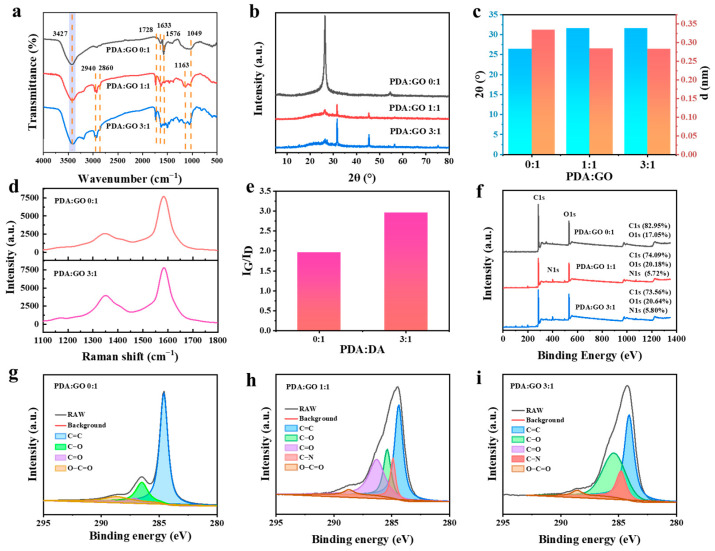
Characterization of rGO from different proportions of PDA to GO: (**a**) FTIR; (**b**) XRD; (**c**) 2θ and d values; (**d**) Raman spectroscopy; (**e**) Raman spectral peak intensity ratio (ID/IG); (**f**) XPS total spectrum; (**g**–**i**) high-resolution XPS spectra of C1s.

Figure 2 shows the test results of rGO at different PDA and GO ratios. The functional group changes during GO reduction were analyzed by FTIR (Figure 2a). GO had a stretching vibration peak of -OH at 3427 cm^−1^, which refers to the functional group of -COOH in GO. At 1049 cm^−1^, 1576 cm^−1^ and 1633 cm^−1^, there are C=O vibration peaks, C=O vibration peaks on the carboxyl group and C=C benzene ring vibration peaks [34]. When PDA/GO ratios reached 1:1 or 3:1, two new peaks appeared, which were 2940 cm^−1^ and 2860 cm^−1^, respectively, corresponding to the methylene stretching vibration peak of PDA, which confirmed the existence of PDA [31]. The newly formed peak at 1163 cm^−1^ was attributed to the stretching vibration of the C-N bond. In addition, compared with GO, the red shift of C=O peak in rGO confirms successful functionalization of rGO by PDA [35].

The XRD spectra (Figure 2b) reveal that GO has a larger interlayer spacing than rGO, as evidenced by its lower-angle reflection peak at 26.36°. As shown in Figure 2c, with the increase in reducing agent PDA, some oxygen-containing functional groups in GO were removed, and the low peak of rGO appeared near a higher angle (26.36° to 31.61°). The interlayer spacing decreased from, 0.334 nm to 0.283 nm [36]. With the increase in PDA content, the reduction degree of GO increased. Figure 2d displays the Raman spectra of GO and rGO, respectively. The main peaks are the G peak (about 1580 cm^−1^) and the D peak (about 1350 cm^−1^) [37]. Compared with GO, the intensity of the D peak of rGO increased gradually (Figure 2d), and the intensity of G peak was basically unchanged. The two-peak intensity ratio (ID/IG) represents the defect density (Figure 2e). When PDA/GO was 0:1 and 3:1, ID/IG was 1.96 and 2.96, respectively. The ratio increased, which proved that the defect density of rGO increases [38], indicating that a large number of oxygen-containing functional groups of GO are removed in the reduction process [39,40], the sp^3^ hybrid carbon atoms are deoxidized, and a new sp^2^ hybrid region is formed again, which is more conducive to electron transport [41]. In Figure 2f, the percentage of C, O, and N elements was obtained in the wide scan XPS spectra of GO and rGO samples. The elemental composition of GO includes C and O with atomic percentages of 82.95 wt% and 17.05 wt%, respectively. With the increase in the ratio of reducing agent PDA to GO, the content of C decreases with the increase in PDA content. At the same time, because of the presence of the N element and a large amount of the O element produced by PDA in the reducing agent, the content of C decreases. Further, more detailed information of functional groups was obtained from the high-resolution C 1s XPS spectra of GO and rGO, where Figure 2g–i is the XPS spectra of GO (PDA/GO = 0:1), rGO (PDA/GO = 1:1), and rGO (PDA/GO = 3:1), respectively. It could be seen from the total spectrum that the N element appeared in the rGO spectrum, and its weight increased by 5.72 wt% and 5.80 wt%, respectively. The content of the C element decreased to 74.09 wt% and 73.56 wt%. The XPS C1s spectra of GO could be divided into four peaks, and the binding energies were about 284.6 eV, 286.5 eV, 287.0 eV, and 288.7 eV, corresponding to C=C, C-O, C=O, and O-C=O in the aromatic ring, respectively [42]. Compared with the former, the XPS C1s spectrum of rGO increases the C-N peak at 284.8 eV [43]. XPS successfully verified that DA successfully reduced GO to rGO and modified its surface. The formation of rGO through the functionalization of GO with PDA made the dispersion of rGO more uniform in aqueous solution, which was conducive to the distribution of rGO in hydrogels and provided more active sites, which was conducive to the formation of interaction [44,45]. In addition, the defect degree of rGO was increased, which was more conducive to the transmission of electrons.

### 2.2. Characteristic of rGO@PVA-PAA Composite Hydrogels

**Figure 3:**
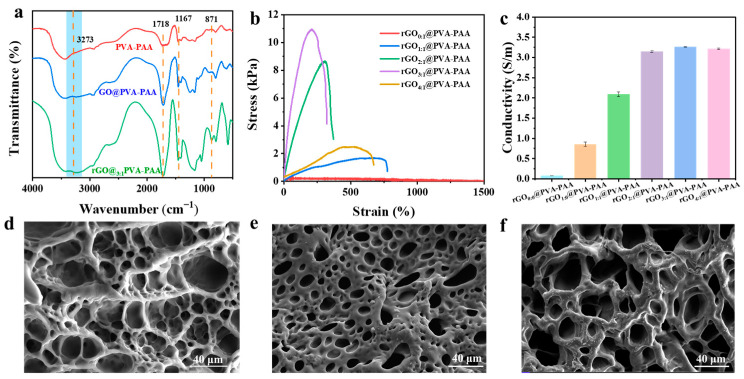
Hydrogels characterization: (**a**) FTIR of PVA-PAA, GO@PVA-PAA and rGO_3:1_@PVA-PAA hydrogels; (**b**) The tensile stress–strain curve and (**c**) electrical conductivity of hydrogels with different PDA/GO ratios; The SEM of (**d**) rGO_2:1_@PVA-PAA, (**e**) rGO_3:1_@PVA-PAA, and (**f**) rGO_4:1_@PVA-PAA hydrogels.

Figure 3a shows the infrared spectrum test of PVA-PAA, GO@PVA-PAA, and rGO_3:1_@PVA-PAA hydrogels. The peak at 3273 cm^−1^ is the absorption vibration of -OH [46], and the energy band of 1718 cm^−1^ is C=O. The peak at 1457 cm^−1^ is related to the stretching vibration peak of C=C; the energy band of 1167 cm^−1^ corresponds to the stretching vibration peak of C-O [47]. The broad absorption band around 871 cm^−1^ was the C=C characteristic peak in the R_1_CH=CH_2_ group. Compared with the PVA-PAA hydrogels, the OH peaks of the hydrogels added with GO (GO@PVA-PAA and rGO_3:1_@PVA-PAA hydrogels) significantly blue shifted, which proved that GO and rGO could have hydrogen bond interaction with PVA or PAA [48]. The C=C double bond became sharper, mainly from GO or rGO, demonstrating its successful addition to hydrogels.

The mechanical properties of rGO@PVA-PAA hydrogels were tested to determine the effect of different PDA/GO ratios on the mechanical properties of hydrogels. Figure S1 was a picture of the hydrogel stretching, which showed that the hydrogel could be stretched to 4.43 times the initial length. As shown in Figure 3b, the maximum tensile strength of the hydrogel exhibits a trend of initial increase followed by a decrease with the increasing PDA/GO ratio. When the ratio of PDA to GO was 3:1, the tensile strength reached its maximum value of 10.95 kPa. This is attributed to the elevated surface functional groups on the functionalized rGO due to the increased PDA/GO ratio, which enhances the hydrogen bonding interactions among PVA, PAA, and rGO, thus improving the tensile strength of the hydrogel [49]. When PDA/GO is 4:1, the concentration of PDA is too high, and the initiator APS will be preferentially reacted when the hydrogel is polymerized, which will reduce the crosslinking degree of the hydrogel and lead to the decrease in the maximum tensile strength of the hydrogel [50]. With the increase in the PDA/GO ratio, the conductivity of the rGO@PVA-PAA hydrogel exhibited an initial increase followed by a stabilizing trend (Figure 3c). The rGO_3:1_@PVA-PAA hydrogel demonstrated the highest conductivity, reaching 3.26 S m^−1^, which was 46.57 times that of the rGO_0:0_@PVA-PAA hydrogel. The significant enhancement in conductivity of the hydrogel can be attributed to the presence of rGO. Firstly, rGO possesses a high specific surface area and a two-dimensional honeycomb-like carbon structure, allowing for good dispersion within the hydrogel and interconnecting to form continuous conductive pathways, which facilitates electron transport [28,51]. Secondly, the high-water content of the hydrogel provides conditions for ion migration. rGO can bind to PVA and PAA in the hydrogel through hydrogen bonding, optimizing interface contact and reducing resistance to electron and ion transport [52]. Consequently, the formed “ion-electron” synergistic conduction mechanism significantly improves the conductivity of the hydrogel.

In addition, through SEM, we found that compared with rGO_2:1_@PVA-PAA hydrogel (Figure 3d), the SEM of rGO_3:1_@PVA-PAA hydrogel (Figure 3e) showed a decrease in pore size and a thickening of pore wall. However, the SEM of rGO_4:1_@PVA-PAA hydrogel (Figure 3f) showed that the pore size increased and the pore wall decreased. This indicates that the increase in PDA/GO ratio is beneficial to the modification of rGO, but the high concentration of PDA reduces the crosslinking degree of hydrogel, thus showing the increase in pore size [50]. Furthermore, EDS point scanning was performed on hydrogel, as shown in Figure S2. EDS showed that the content of the C element in the rGO_3:1_@PVA-PAA hydrogel was the highest one, up to 66.12 wt%. The proportion of N element was 1.94 wt%, which proved that rGO was successfully incorporated into the hydrogel.

### 2.3. Mechanical Properties and Conductivity of rGO@PVA-PAA Hydrogels

**Figure 4:**
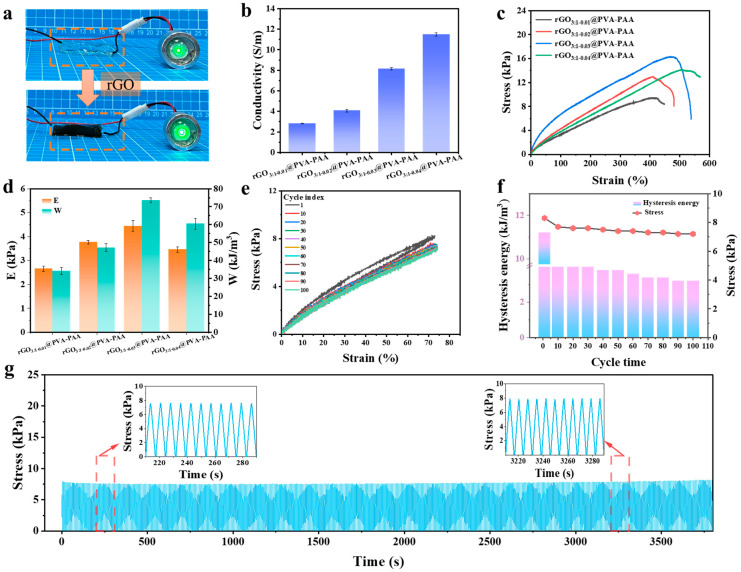
Hydrogel properties: (**a**) The light bulb brightness was observed in the closed loop of PVA-PAA and rGO@PVA-PAA; (**b**) the conductivity of hydrogels with different rGO contents; (**c**) tensile stress–strain curves, (**d**) elastic modulus and toughness of hydrogels with different rGO contents; (**e**) tensile loading–unloading stress–strain curve; (**f**) hysteretic energy; (**g**) at 100% strain, the hydrogel was subjected to 500 cycles of tensile loading and unloading.

As a conductive filler, rGO had a great influence on the electrical properties of hydrogels. As shown in Figure 4a, upon the incorporation of rGO, the color of the hydrogel changed from transparent to black, and the brightness of a small bulb increased. Preliminary inference suggests that rGO may positively enhance the conductivity of the hydrogel by forming conductive pathways within its network.

Subsequently, the conductivity of the hydrogel was tested. The electrical conductivity increased with the increase in rGO, from 2.81 S m^−1^ to 11.49 S m^−1^ (Figure 4b). This was due to the increase in rGO content and the formation of more conductive channels in the hydrogel [53,54]. To investigate the impact of various rGO content on the mechanical properties of hydrogels, mechanical tensile tests were conducted on the hydrogels. As shown in Figure 4c, when the content of rGO increased from 0.01 g to 0.03 g, the tensile strength of the hydrogels rose from 11.85 kPa to 20.38 kPa. However, as the rGO content further increased, a decrease in tensile strength was observed, primarily attributed to excessive rGO leading to partial agglomeration within the hydrogel matrix [50]. Consistent trends were noted in both the elastic modulus and toughness, with the rGO_3:1-0.03_@PVA-PAA hydrogel exhibiting an elastic modulus of 4.24 kPa and a toughness of 72.11 kJ m^−3^ (Figure 4d). Through mechanical and electrical investigations, we found that the rGO_3:1-0.03_@PVA-PAA hydrogel exhibited optimal comprehensive performance. In addition, the hydrogel was tested by a tensile loading–unloading cycle. From Figure 4e,f, it can be seen that the stress of the hydrogel gradually decreases and then basically keeps stable. The hysteretic energy also exhibits the same variation law; the first load-to-unload cycle was 11.23 kJ m^−3^, and the subsequent cycle was basically stable at 9.84 kJ m^−3^. This is because during the first tensile loading–unloading cycle, reversible crosslinks are formed between the hydroxyl groups (-OH) on PVA and the carboxylic acid groups (-COOH) on PAA in the hydrogel through hydrogen bonding. During the tensile process, partial breakage of these weak hydrogen bonds leads to energy dissipation, which fails to recover promptly in subsequent cycles [55]. As the number of cycles increases, the breakage and reformation of these non-covalent interactions gradually stabilize, resulting in a corresponding stabilization of hysteresis energy [56]. Furthermore, the introduction of rGO enhanced the cross-linking degree among the scaffolds [40], providing additional physical support and a stress dispersion mechanism to prevent localized stress concentration. This contributed to reducing energy loss during subsequent cycles by mitigating potential points of failure [52].

Next, to further investigate its stability and fatigue resistance, the rGO_3:1-0.03_@PVA-PAA hydrogel underwent 500 consecutive tensile loading–unloading cycles. As illustrated in Figure 4g, under a strain of 100%, the cyclic tensile curves displayed no significant displacement or rupture, demonstrating excellent elasticity and shape recovery performance of the rGO_3:1-0.03_@PVA-PAA hydrogel.

### 2.4. Adhesion Property of rGO_3:1-0.03_@PVA-PAA Hydrogel

**Figure 5:**
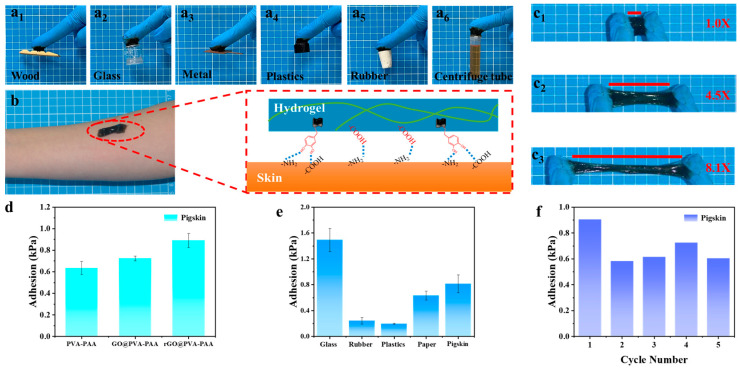
Adhesion ability of rGO_3:1-0.03_@PVA-PAA hydrogel: (**a_1_**–**a_6_**) Photos of adhesion of hydrogel to various materials; (**b**) adhesion mechanism of hydrogels; (**c_1_**–**c_3_**) stretching photographs of hydrogel adhesion; (**d**) adhesion strength of three hydrogels; (**e**) the adhesion strength of hydrogels on various substrates; (**f**) five adhesion cycles of the hydrogel on pig skin.

As a kind of vibrating sound wave, sound has the highest intensity at the place where it occurs, and its intensity gradually decreases with the passage of distance [57]. Therefore, the adhesion of the flexible sensor at the vocal site is particularly important. Figure 5a_1_–a_6_ shows that hydrogels adhere to the surfaces of wood, inorganic materials (glass, metal), and organic materials (plastics, rubber), respectively, and could even adhere to a centrifuge tube filled with methyl red solution (10 mL). In Figure 5b, taking human skin as an example, the good adhesion of the hydrogel to human skin is due to the interaction of the hydroxyl group on the catechol group with the amino and carboxyl groups on the skin through hydrogen bonding, and the carboxyl group in PAA interacted with the amino group in the skin through hydrogen bonding [58]. These interactions made the hydrogel adhere firmly to the skin. While not damaging the skin or causing pain, it was a superior and more convenient feature than previous strong adhesives. In addition, the hybrid structure of PDA could interact with various contact surfaces to form cation–π interaction or π–π stacking [59], so that rGO@PVA-PAA hydrogels could adhere to various substrate surfaces.

When the hydrogel was attached to both ends of the nitrile glove for stretching (Figure 5c_1_–c_3_), the hydrogel could be stretched from 1 to 8.1 times and still adhere firmly to both ends of the glove. It was proved that the hydrogel has good adhesion and could adhere to the skin effectively, which provides a more powerful condition for sound monitoring. In Figure 5d, the influence of components on the adhesion of hydrogels was discussed. The adhesion strength of PVA-PAA hydrogels was 0.63 kPa, and that of GO@PVA-PAA was 0.72 kPa, which proved that the addition of GO had no effect on the adhesion of hydrogels. The adhesion strength of rGO@PVA-PAA hydrogel was 0.89 kPa, which proved the principle of Figure 5b. The synergistic effect of PAA and PDA improved the adhesion of the hydrogel. As shown in Figure 5e, rGO@PVA-PAA hydrogel exhibited different adhesion strengths on different substrates, such as 1.49 kPa on a glass substrate, 0.24 kPa on a rubber substrate, and 0.20 kPa on a plastics substrate. The adhesion strength on paper substrate was up to 0.63 kPa, and that on the pig skin substrate was 0.82 kPa. It was proved that the hydrogels showed diverse adhesion on different substrates. In addition, the adhesion force of the hydrogel remained above 70% after repeated adhesion on different materials for 5 times, which proved its good repeated adhesion (Figure 5f and Figure S3).

### 2.5. Sensing Performance of rGO_3:1-0.03_@PVA-PAA Hydrogel

**Figure 6:**
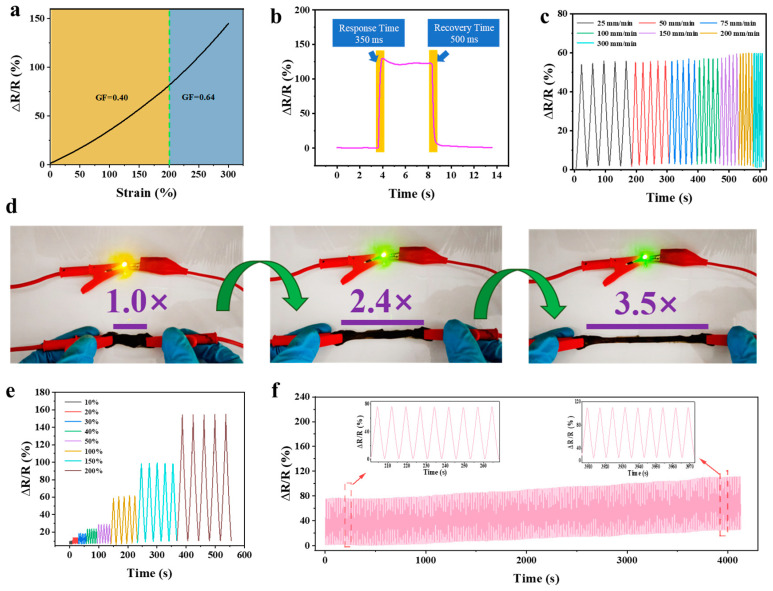
(**a**) Relative resistance change rate of rGO_3:1-0.03_@PVA-PAA hydrogel at different strains; (**b**) response time and recovery time of hydrogel; (**c**) relative resistance change rate of hydrogel at different rates (25, 50, 75, 200, 250, 200, and 300 mm min^−1^); (**d**) photos of light bulb brightness corresponding to hydrogel under different stretching deformations; (**e**) relative resistance change rate of hydrogel at different strains (10, 20, 30, 40, 50, 100, 150, and 200%); (**f**) at 100% strain, the resistance change rate of hydrogel after 500 cycles of tensile loading and unloading was obtained.

Considering the specificity required for sound detection and the type of hydrogel-based sensor being a strain sensor, it was of vital significance to explore the changes of mechanical properties of rGO_3:1-0.03_@PVA-PAA hydrogel in different states for subsequent sound detection. It is proved that rGO_3:1-0.03_@PVA-PAA hydrogel has good mechanical properties and excellent conductivity by mechanical and electrical tests. The sensitivity of the hydrogel to external strain can be evaluated by the linear relationship (GF) between the relative resistance change and the strain [56]. It can be seen from Figure 6a that ΔR/R gradually increases with the expansion of strain. The rate of change can be categorized into two linear response regions: 0–200% with a GF of 0.40 and 200–300% with a GF of 0.63. At the same time, the transient response time and recovery time of the hydrogel were tested by tensile test. The results show that, under a strain of 110%, the hydrogel exhibits a response time of 350 ms and a recovery time of 500 ms (Figure 6b). This underscores the hydrogel’s capacity for rapid strain sensing [60]. Table S1 shows the comparison of sensitivity, detection range, response time, recovery time, and conductivity between the rGO_3:1-0.03_@PVA-PAA hydrogel sensor and other reported hydrogel sensors [36,39,40,44,61,62,63,64,65,66,67]. It can be found that the rGO_3:1-0.03_@PVA-PAA hydrogel sensor has the best comprehensive performance. Furthermore, we examined the relative resistance change rate of the hydrogel under various strain rates. The hydrogel exhibited relatively consistent relative resistance changes across various rates (Figure 6c). This demonstrates that the hydrogel sensor maintains good electrical stability across a wide strain range. Small light bulbs with different power with a fixed voltage of 15 V were selected for the experiment as shown in Figure 6d. As the strain on the hydrogel increased from 10% to 200%, the relative resistance of the hydrogel gradually increased. During five loading–unloading cycles, the tensile strain of the hydrogel was converted into a stable electrical signal (Figure 6e). Fatigue resistance is also an important indicator of the application of the hydrogel sensor [68]. After 500 cycles, the signal of the hydrogel remained stable, indicating good cycle stability and durability, which is conducive to applications in human health monitoring.

### 2.6. rGO_3:3-0.03_@PVA-PAA Hydrogel Based Sensor

**Figure 7:**
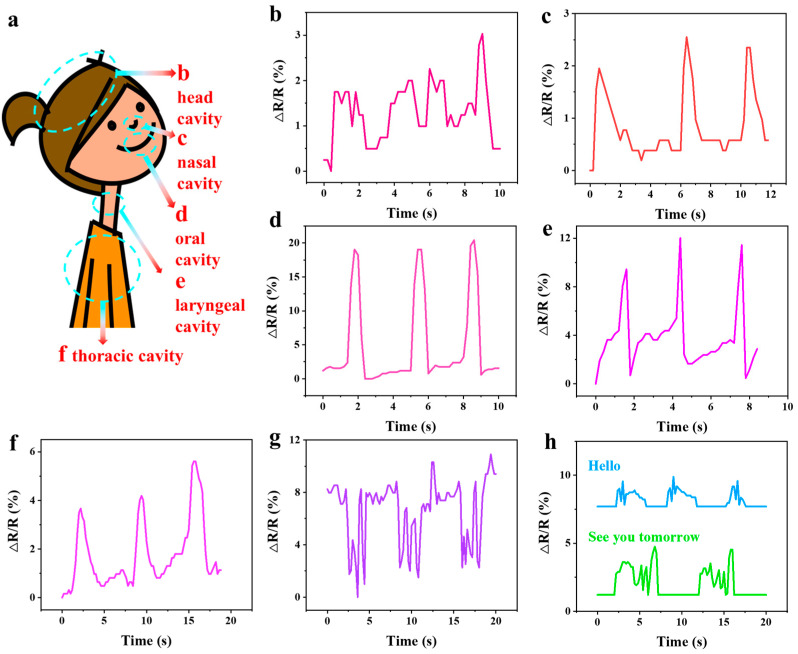
(**a**) Corresponding parts of five-cavity resonance test; (**b**) relative resistance change rate of head cavity voice; (**c**) relative resistance change rate of nasal cavity voice; (**d**) relative resistance change rate of oral cavity voice; (**e**) relative resistance change rate of laryngeal cavity voice (**f**) relative resistance change rate of thoracic cavity voice; (**g**) relative resistance change rate of cough voice; (**h**) actual human vocalization tests.

The hydrogel-based sensor with significant adhesion was adopted to directly adhere to the corresponding parts (Figure 7a) for the test of five-cavity sound. As shown in Figure 7b, the hydrogel-based sensor sensitively detected the slight vibration emitted by the resonance of the head cavity. In addition, obvious relative resistance changes were also detected in the nasal cavity (Figure 7c), oral cavity (Figure 7d), laryngeal cavity (Figure 7e), and thoracic cavity (Figure 7f). The relative resistance change rate of the oral cavity and thoracic cavity is significantly higher than that of the other three cavities, indicating that they are more susceptible to vibration. It was proved that the hydrogel-based sensor was highly responsive, and the signal was highly repeated in each cycle. It could be used as a sound sensor in daily life. In addition, cough, a typical symptom of catching a cold, was also tested. As shown in Figure 7g, monitoring of the laryngeal cavity was conducted on the volunteers. With three repeated coughs as the signal, coughing and wheezing after coughing could be clearly distinguished. To prove its use in everyday life, a further test of normal speech in the larynx was carried out. When the volunteers say “Hello” and “See you tomorrow”, respectively, as shown in Figure 7h, the hydrogel-based sensor could clearly monitor this information from the syllable or sentence, and each expression had its own independent signal, indicating that the sensor had a broad background in voice monitoring. At the same time, excellent adhesion ensured that the hydrogel-based sensor had a reliable detection effect in some large human movements (Figure S4). The hydrogel-based sensor could accurately and repeatedly monitor the real-time movements of human fingers, elbows, and wrists and accurately capture the motion state of the pauses and re-movements of these parts. When the hydrogel-based sensor was combined with the electrocardiograph (Figure S5), its signal could be accurately captured [69]. Compared with the clinic used iron patch of the electrocardiograph, the curve measured by the hydrogel-based sensor was consistent with that of the electrocardiograph, and the signal distortion would not occur [70]. At the same time, due to its self-adhesive, hydrogel-based ECG detection probes, with a modulus close to that of skin, are soft, comfortable, and close to body temperature, making them more acceptable to patients; therefore, the fear and discomfort caused to patients by the use of metal probes, which often occurs clinically, can be avoided. The hydrogel-based sensor showed its advantage in writing recognition, clearly recognizing letter signals (Figure S4), indicating wide potential in human–computer interaction.

**Figure 8:**
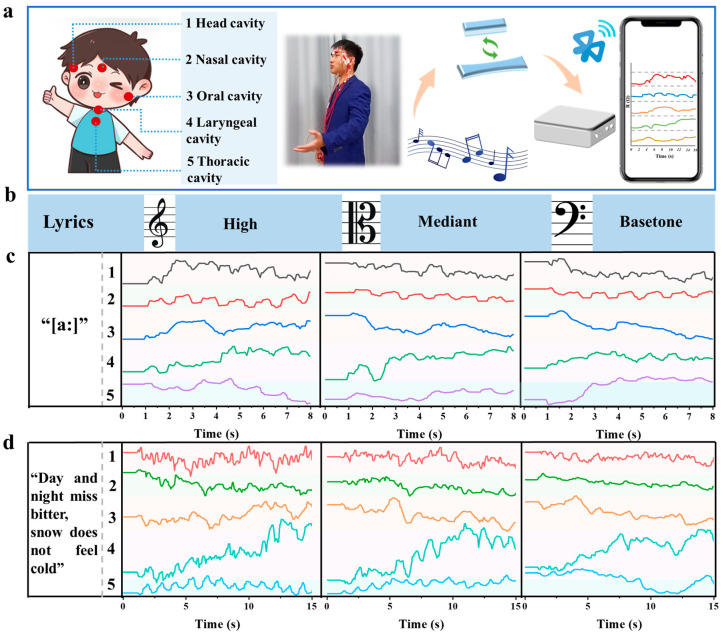
(**a**) A schematic illustration of the mechanism of multi-channel hydrogel sensors to monitor the synergetic vibration at multiple sites during a singer’s performance; (**b**) detection of the vocal patterns of a singer’s high, medium, and low pitches, respectively; (**c**) signals obtained from multi-site synergetic monitoring of the five vocal cavities during the singing of a single syllable; (**d**) signals obtained from multi-site synergetic monitoring during the singing of a complete song.

Previously, multiple sites on a singer’s body during performance were detected using single-channel hydrogel sensors, and the results demonstrated that the prepared sensors were capable of detecting subtle vibrations of the human body. To further understand the relationships among these detected signals, multi-channel hydrogel sensors capable of multi-site synergetic detection were subsequently developed. These sensors were employed to track the synchronous changes in the head cavity, nasal cavity, oral cavity, laryngeal cavity, and thoracic cavity of singers when high, medium, and low pitches were sung, thereby allowing for better distinction of the vocal patterns of these different pitches. Self-adhesive hydrogel patches were in favor of fixing onto the five vocal sites of the human body, and the relative resistance change signals from the hydrogel patches during vocalization were collected and integrated onto a portable workstation. These signals were then converted into digital signals, wirelessly transmitted via Bluetooth, and displayed on a mobile phone screen, as illustrated in Figure 8a and Movie S1.

As shown in Figure 8b,c, real-time monitoring was first conducted on a singer sustaining single syllables [a:] across the dimensions of high, medium, and low pitches. During the singing of high pitches, it was observed that resonance signals from the head cavity, nasal cavity, and oral cavity were relatively prominent, while signals from the laryngeal cavity were weaker, indicating that the head and nasal cavities were crucial for singers to produce high pitches. When medium pitches were sung, signals from the head cavity, nasal cavity, and thoracic cavity were relatively flat and low, with resonance characteristics mainly concentrated in signals from the oral and laryngeal cavities. When low pitches were sung, the thoracic cavity resonance signals were more significant. Consequently, signals were collected from a singer singing a complete song using high, medium, and low pitch techniques as shown in Figure 8d. Compared to single-syllable vocalizations, due to changes in consecutive syllables, jumps in pitch frequency, and increases in vocal intensity, the characteristics of signal fluctuations remained similar, while vocal signals from multiple cavities were significantly enhanced and more distinguishable. The fast Fourier transform (FFT) was further used for signal processing and analysis of the audio, resulting in frequency domain spectrograms of the audio signals [71]. As shown in Figure S6a, when a singer performed a song, the frequencies of high-pitched signals are mainly concentrated in the high-frequency range (600–800 Hz), with the highest amplitude intensity reaching 3375. This indicates that high-pitched signals possess higher frequency components and a relatively concentrated energy distribution. The frequencies of mid-pitched signals are primarily distributed in the mid-frequency range (400–600 Hz), with the highest amplitude intensity reaching 2295, and their energy distribution is relatively broad (Figure S6b). The low-frequency range (100–300 Hz) is where low-pitched signals are mainly distributed, with the highest amplitude intensity reaching 1380 (Figure S6c). It is evident that there are significant differences in frequency and energy distributions among high-, mid-, and low-pitched signals. These differences suggest that the detected signals of high, mid, and low pitches from singers are distinguishable, further demonstrating the reliability of the monitoring results obtained through the five-cavity sensing mechanism. In summary, the prepared multi-channel hydrogel sensors exhibited superior sensing capabilities for the complex, abundant, and nuanced vocalizations of singers. The developed multi-site voice-coordinated detection hydrogel sensors are held to have great potential in health monitoring and skill training.

## 3. Conclusions

This work presents a high-performance rGO@PVA-PAA composite hydrogel sensor for multi-site, high-fidelity sound detection and physiological signal monitoring, inspired by the five-vocal-cavity resonance mechanism. The hydrogel was synthesized using a one-pot strategy, integrating a dual-network framework (PVA as the first network and PAA as the second network) with PDA-derived rGO as conductive fillers. The redox reaction between PDA and GO facilitated uniform rGO dispersion within the hydrogel matrix, achieving exceptional electrical conductivity (8.15 S m^−1^) and rapid response time (350 ms). Synergistic interactions between PDA and PAA further enhanced the hydrogel’s robust skin-adhesion strength (0.89 kPa), enabling conformal attachment for reliable signal acquisition. The sensor demonstrated excellent transmissibility, remarkable sensitivity, and durability across 500 load–unload cycles. Aimed at the sound detection with the features of high frequency and low amplitude, the hydrogel could effectively detect the information from the synergistic vibration of five cavities at the same time. Especially, it can be used in the healthcare area, including symptom diagnosis by identifying the information of cough and electrocardiogram detection. In addition, the prepared hydrogel can also be utilized in human–computer interaction fields such as information writing. The sound detection sensing technology inspired by the five-chamber resonance phenomenon presented in this article is expected to provide new insights for the development of flexible wearable electronic devices.

## 4. Materials and Methods

### 4.1. Materials

Polyvinyl alcohol (PVA, polymerization degree of 1750 ± 50) and hydrochloric acid (HCl, 36%) were purchased from Sinopharm Chemical Reagent Co., Ltd. (Shanghai, China). Ammonium persulfate (APS) was purchased from Tianjin Fuchen Chemical Reagent Co., Ltd. (Tianjin, China). Graphene oxide (GO, >99%) was purchased from Aladdin Chemical Reagent Co., Ltd. (Shanghai, China). Acrylic acid (AA, >99%) and dopamine hydrochloride (DA, >99%) were purchased from Shanghai Macklin Biochemical Technology Co., Ltd. (Shanghai, China).

### 4.2. Preparation of rGO@PVA-PAA Composite Hydrogel

Firstly, 0.05 g of GO powder was weighed and dispersed in 10 mL of deionized water in a 100 mL beaker. The mixture was then sonicated for 1 h. PDA oligomer was obtained through the oxidation reaction of DA for 20 min under stirring in an air atmosphere in 10 mL of Tris-HCl aqueous solution at pH 8.5. Subsequently, the GO solution was added to the PDA solution. After further stirring the mixture for 1 h, rGO was obtained. The PDA/rGO mixture was centrifuged at 10,000 rpm for 10 min, and this procedure was repeated three times. The resultant sediment was redispersed in clean water, transferred to a refrigerator, and frozen at −27 °C for 12 h. Following this, freeze-drying was performed at −50 °C for 48 h to obtain rGO powder.

After the GO was reduced to rGO, 0.2 g of PVA was dissolved in deionized water at 95 °C for 2 h to prepare a 2 wt% PVA solution. A 4 mL aqueous solution of AA containing the initiator APS (at 1.0 wt% with respect to AA monomer) was mixed with 10 mL of PVA solution for 5 min under N_2_ bubbling to eliminate oxygen. Subsequently, 10 mL of the PDA-rGO solution was added to the 14 mL mixture of PVA-AA. The combined solution was stirred for 20 min, and then the reaction vessel was transferred to a 70 °C water bath for further polymerization for 4 h. The resultant hydrogel was labeled as rGOx-y@PVA-PAA, where x indicates the mass ratio of PDA to GO and y indicates the content of GO. For example, rGO_3:1-0.03_@PVA-PAA refers to the hydrogel sample prepared with a mass ratio of PDA to GO of 3:1 and a GO weight of 0.03 g.

### 4.3. Characterization

All hydrogel samples were pre-frozen at −20 °C for 24 h and then freeze-dried at 50 °C for 48 h. The dried hydrogel samples were observed using a field emission scanning electron microscope (SEM, Merlin Compact, Carl Zeiss, Oberkochen, Germany) under conditions of an accelerating voltage of 5 kV and a working distance of 6.5 mm. Under conditions of an accelerating voltage of 15 kV and a working distance of 6.5 mm, the elemental composition and distribution were analyzed by EDS. The structural composition of the hydrogel samples was measured by a Fourier transform infrared spectrometer (FTIR, iS50, Thermo Fisher Scientific, Waltham, MA, USA) in the wavelength range of 400–4000 cm^−1^. The X-ray diffraction (XRD) pattern of the graphene oxide sample powder was obtained using an X-ray powder diffractometer (D8 ADVANCE, Bruker, Germany). The scanning range was 5–80°, and the scanning time was 0.1 s for each point. The elemental composition and reduction degree of graphene oxide were analyzed by X-ray photoelectron spectroscopy (XPS, Thermo Scientiffc K-Alpha+, Thermo Fisher Scientiffc, Waltham, USA). A Raman analytical instrument (Bidtech, BWS465-532H) was used to analyze the molecular structure of GO.

### 4.4. Mechanical Property

The hydrogel specimens were cut into cuboids at the size of 40 mm × 10 mm × 5 mm. The mechanical test was carried out via the microcomputer-controlled electronic universal material testing machine (Hy-0580, Shanghai Heng Wing-Instrument Co., Ltd., Shanghai, China). The tensile stress was defined as the load divided by the original sectional area of the specimen. The tensile strain was defined as the deformation height divided by the original height of the specimen. The hydrogel specimen was cut into cuboids at the size of 40 mm × 10 mm × 5 mm before the tensile loading–unloading experiment. The hydrogel was subjected to 500 tensile loading and unloading cycles at 100% strain. The tensile rate was set at 100 mm min^−1^.

### 4.5. Conductivity Measurement

The hydrogel, with dimensions of 40 mm × 10 mm × 6 mm, is connected to a small bulb via a wire in the circuit. By observing the brightness of the bulb, an initial assessment of the hydrogel’s conductivity is made. The upper and lower sides of the hydrogel sample with a size of 30 mm × 30 mm × 5 mm were covered with copper sheets as electrodes so that the electrodes were completely bonded to the hydrogel sample. Subsequently, the electrodes are connected to an electrochemical workstation (Ivium Vertex. C. EIS, The Netherlands Vertex. C. EIS, Ivium Technologies BV Co., Ltd., Eindhoven, The Netherlands) with a wire, and the electronic conductivity of the hydrogel sample was measured by the electrochemical impedance method within a frequency range of 0.1 to 105 Hz. The same sample was tested three times, and the average value and error range were calculated. The entire test is conducted at a room temperature of approximately 25 °C and a relative humidity of approximately 50%. The electronic conductivity is calculated as follows:

(1) σ=D/(S×R) 

where *σ* is the electronic conductivity (S m^−1^), *D* is the distance between the two electrodes (m), *S* is the effective area of the hydrogel electrolyte (m^2^), and *R* is the resistance of the hydrogel (Ω).

### 4.6. Adhesion Property

The hydrogel was cut into slices with a cross-section of 30 mm × 10 mm, and the two substrates (glass, plastic, paper, and pig skin) were used as the support to construct a single bypass structure, which was kept unchanged at room temperature for 10 min. The two ends of the glass sheet will be stretched on a universal test machine to obtain adhesion. Each sample was tested three times and averaged. The adhesion strength is calculated as the adhesion force divided by the cross-sectional area of the hydrogel.

### 4.7. Fabrication of the Flexible Strain Sensor

The hydrogel was utilized as a conductive material for the fabrication of flexible sensors. A cuboid-shaped hydrogel with dimensions of 30 mm × 10 mm × 5 mm was cut from the bulk material. To establish an external circuit, two wires were connected to opposite sides of the hydrogel. The electrochemical workstation was preset to the I-t (current-time) mode, where a constant voltage of 1 V was applied, and the real-time current was recorded during the discharge process. The formula used to calculate the relative resistance change rate is as follows:

(2) $$∆R/R=(R-R_{0})/R_{0}\;$$

where *R* and *R*_0_ represent the pristine resistance (Ω) and the real-time resistance (Ω), respectively. An electrochemical workstation and a universal tensile testing machine were used together to test the performance of the hydrogel sensor. The experimental parameters of the universal tensile testing machine, such as 4.4 mechanical property settings, were consistent. To detect human motion, the hydrogel was attached to various parts of the body. Subsequently, both ends of the hydrogel were connected to an electrochemical workstation via a copper wire, enabling the recording of resistance changes. In terms of sound detection, the prepared hydrogel-based sensors were affixed to the head cavity, nasal cavity, mouth cavity, throat cavity, and chest cavity, respectively, to monitor the vibration of five cavities, in which the throat cavity can also detect speech and cough. The vibration of the five parts when singing high, medium, and low is constructed by the wireless multi-channel signal transmission module (LinkZill, LZ-01ARC, Hangzhou, China). The participant for the wearable experiment is the first author of this article, and this case does not need the approval from the Ethics Committee of the University of Jinan.

## Data Availability

The original contributions presented in this study are included in the article/Supplementary Materials. Further inquiries can be directed to the corresponding authors.

## Supplementary Materials

The following supporting information can be downloaded at: https://www.mdpi.com/article/10.3390/gels11040233/s1, Table S1: This work is compared with other work-related performance. Figure S1: Photos of hydrogel stretching process; Figure S2: EDS of rGO_3:1_@PVA-PAA; Figure S3: Five adhesion cycles of the hydrogel on (a) glass, (b) rubber, (c) plastic, and (d) paper. Figure S4: Application of hydrogel in human motion monitoring and writing board: (a) schematic diagram of hydrogel sensor connected with computer; (b) Swallowing; (c) Finger bending; (d) Elbow bending; (e) Finger bending and static transition; (f) Wrist bending; (g) Hydrogel writing board schematic diagram; hydrogel writing (h) A, (i) B, (j) C. Figure S5: Hydrogel combined with electrocardiograph: (a) Distribution of hydrogel-based electrodes on human body; (b) Normal electrocardiogram; Electrocardiogram measured with(c_1_) metal and (c_2_) hydrogel electrodes; Figure S6: The frequency domain spectrum of the singer’s (a) high, (b) medium, and (c) low sound audio signals after FFT processing. Movie S1: Real-time signal output of multi-channel sensing equipment during volunteer singing.

## Abbreviations

AA	Acrylic acid
APS	Ammonium persulfate
DA	Dopamine hydrochloride
EDS	Energy-dispersive spectrometer
FTIR	Fourier transform infrared spectrometer
GO	Graphene oxide
HCI	Hydrochloric acid
PVA	Polyvinyl alcohol
PDA	Polydopamine
PAA	Polyacrylic acid
rGO	Reduced graphene oxide
SEM	Scanning electron microscope
XRD	X-ray diffraction
XPS	X-ray photoelectron spectroscopy
