# Supplementary material for: Five-Cavity Resonance Inspired, rGO Nano-Sheet Reinforced, Multi-Site Voice Synergetic Detection Hydrogel Sensors with Diverse Self-Adhesion and Robust Wireless Transmissibility
Source: Gels. 2025 Mar 23;11(4):233. doi: 10.3390/gels11040233 (PMC12027062; doi:10.3390/gels11040233)
Supplement: Supplementary file 1 [file gels-11-00233-s001.zip › Supporting Information.pdf]

Supporting Information

Five-Cavity Resonance Inspired, rGO Nano-Sheet Reinforced, Multi-Site Voice Synergetic Detection Hydrogel Sensors with Diverse Self-Adhesion and Robust Wireless Transmissibility

Yue Wu [1,2], Kewei Zhao [2,3], Jingliu Wang [1,2], Chunhui Li [2], Xubao Jiang [1,*], Yudong Wang [4,*] and Xiangling Gu [2,*]

1 College of Chemistry and Chemical Engineering, University of Jinan, Jinan 250024, China

2 School of Health and Medicine, Dezhou University, Dezhou 253023, China

3 College of Chemistry and Chemical Engineering, Shandong University of Technology, Zibo 255000, China

4 College of Biological and Chemical Engineering, Guangxi University of Science & Technology, Liuzhou 545006, China

Table S1 This work is compared with other work-related performance.

Sample	Sensitivity	Detection range (%)	Response time (ms)	Recovery time (ms)	Conductivity (S/m)	Reference
**$rGO_{3:1-0.03}$@PVA-PAA**	0.65	0-300	350	500	8.15	This work
**PDA-rGO/PVA**					0.87	[36]
**rGO/CMCNa/PAA**	1	0-300	187		0.56	[39]
**rGO/PDA-Gly-PVA**	2.78	0-200	370	260	2.3	[40]
**PVA-prGO-PDA**					0.005	[48]
**L-PAA-OH**	0.32	0-300	116	68	0.61	[61]
**SbQ/SA/$FeCl_3$/Gly**	0.61	0-100			0.38	[62]
**SA/PAm/Ga**	2.59	0-100	110	90	1.9	[63]
**PVA-M-H (//)**	1.17	0-100	300	300	0.38	[64]
**$AlCl_3$/P(AM-co-MMA)**	0.41	0-100	300			[65]
**PCNAT-Fe**	0.75	0-50	960	1080	6.62	[66]
**AgNSs/PVA**	1.87	0-50	609	582		[67]

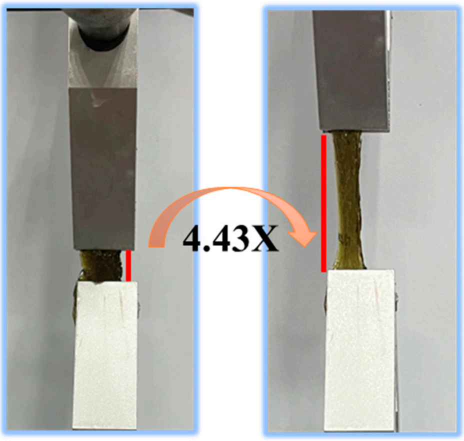

Figure S1. Photos of hydrogel stretching process.

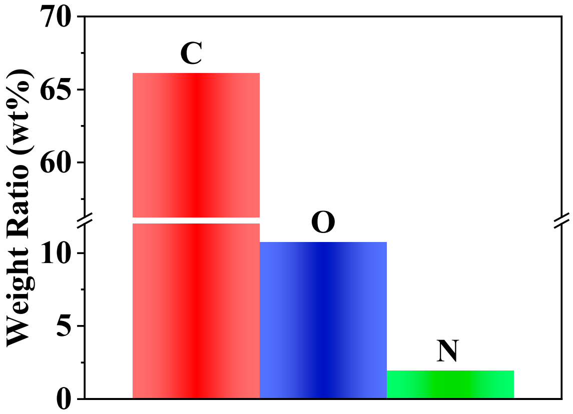

Figure S2. EDS of $rGO_{3:1}$@PVA-PAA.

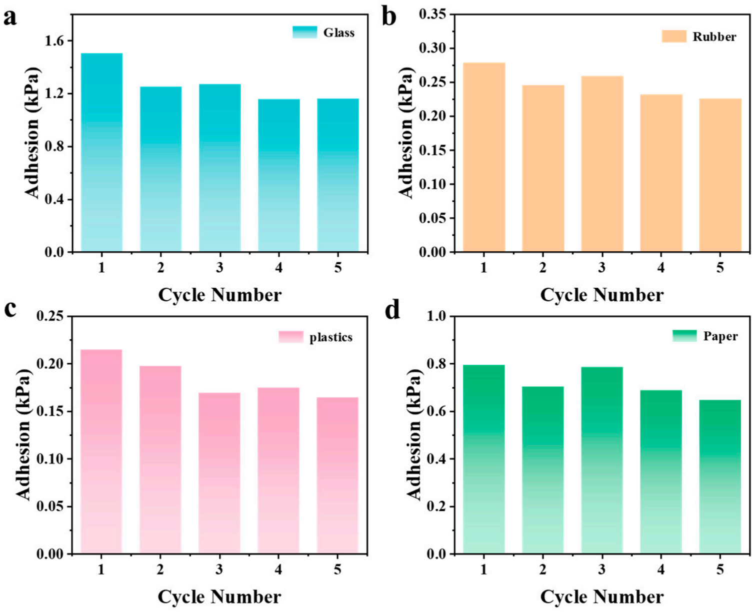

Figure S3. Five adhesion cycles of the hydrogel on (a) glass, (b) rubber, (c) plastic and (d) paper.

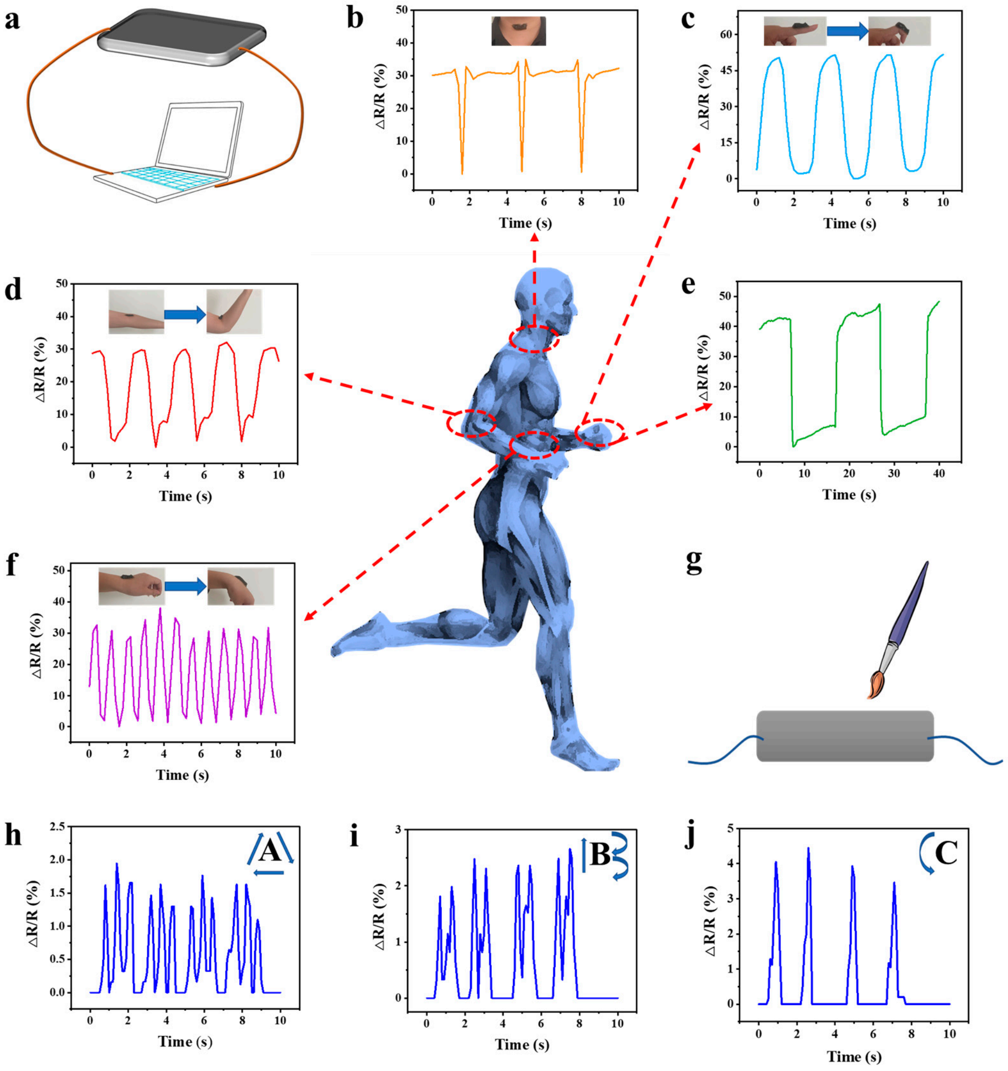

Figure S4. Application of hydrogel in human motion monitoring and writing board: (a) schematic diagram of hydrogel sensor connected with computer; (b) Swallowing; (c) Finger bending; (d) Elbow bending; (e) Finger bending and static transition; (f) Wrist bending; (g) Hydrogel writing board schematic diagram; hydrogel writing (h) A, (i) B, (j) C.

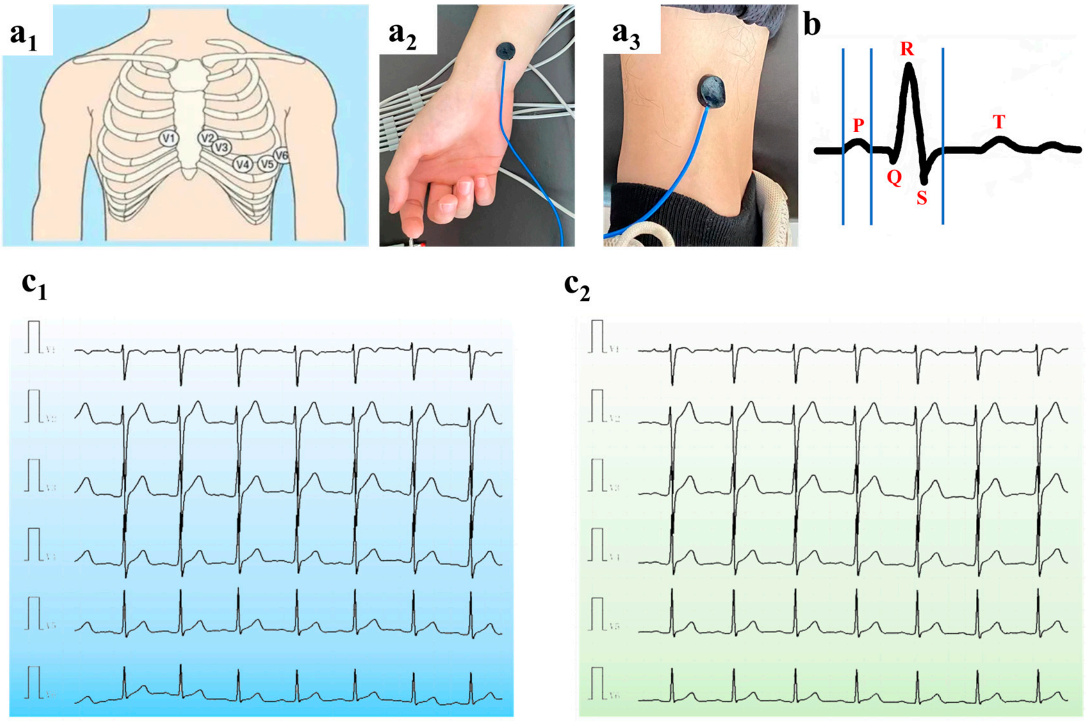

Figure S5. Hydrogel combined with electrocardiograph: (a) Distribution of hydrogel-based electrodes on human body; (b) Normal electrocardiogram; Electrocardiogram measured with (c_1) metal and (c_2) hydrogel electrodes.

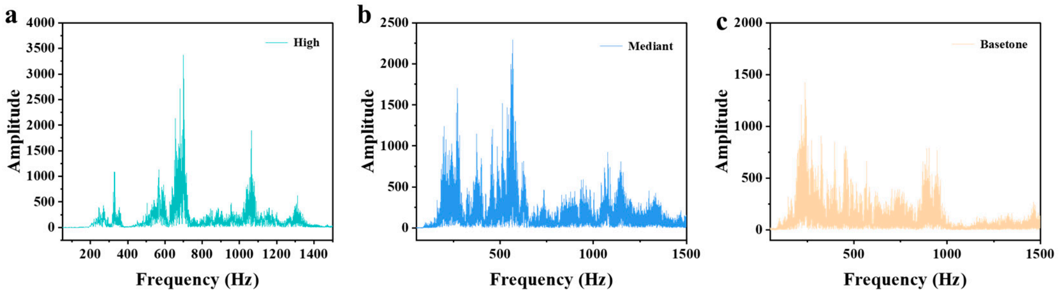

Figure S6. The frequency domain spectrum of the singer 's (a) high, (b) medium and (c) low sound audio signals after FFT processing.

Movie S1: Real-time signal output of multi-channel sensing equipment during volunteer singing.
